# Low Intensity Ultrasound as an Antidote to Taxane/Paclitaxel-induced Cytotoxicity

**DOI:** 10.7150/jca.71263

**Published:** 2022-04-18

**Authors:** Celina Amaya, Elizabeth R. Smith, Xiang-Xi Xu

**Affiliations:** 1 Department of Radiation Oncology, University of Miami Miller School of Medicine, Miami, FL 33136; 2 Department of Obstetrics, Gynecology and Reproductive Science, University of Miami Miller School of Medicine, Miami, FL 33136; 3 Sylvester Comprehensive Cancer Center, University of Miami, Miller School of Medicine, 1120 NW 14th Street, Miami, FL, USA

**Keywords:** Ultrasound, shock wave, microtubules, Taxol/paclitaxel, cytotoxicity, peripheral neuropathy, alopecia, neutropenia, cancer chemotherapy, side effects

## Abstract

The taxane family of compounds, including Taxol/paclitaxel and Taxotere/docetaxel, are surprisingly successful drugs used in combination or alone for the treatment of most major solid tumors, especially metastatic cancer. The drugs are commonly used in regimen with other agents (often platinum drugs) as frontline treatment, or used as a single agent in a dose dense regimen for recurrent cancer. The major side effects of taxanes are peripheral neuropathy, alopecia, and neutropenia, which are grave burden for patients and limit the full potential of the taxane drugs. Especially in the current treatment protocol for peripheral neuropathy, taxane dosage is reduced once the symptoms present, resulting in the loss of full or optimal cancer killing activity.

Substantial efforts have been made to address the problem of cytotoxic side effects of taxanes, though strategies remain very limited. Following administration of the taxane compound by infusion, taxane binds to cellular microtubules and is sequestered within the cells for several days. Taxane stabilizes and interferes with microtubule function, leading to ultimate death of cancer cells, but also damages hair follicles, peripheral neurons, and hemopoietic stem cells. Currently, cryo-treatment is practiced to limit exposure and side effects of the drug during infusion, though the effectiveness is uncertain or limited.

A recent laboratory finding may provide a new strategy to counter taxane cytotoxicity, that a brief exposure to low density ultrasound waves was sufficient to eliminate paclitaxel cytotoxicity cells in culture by transiently breaking microtubule filaments, which were then relocated to lysosomes for disposal. Thus, ultrasonic force to break rigid microtubules is an effective solution to counter taxane cytotoxicity. The discovery and concept of low intensity ultrasound as an antidote may have the potential to provide a practical strategy to counter paclitaxel-induced peripheral neuropathy and alopecia that resulted from chemotherapy.

Taxanes are a class of important drugs used in chemotherapy to treat several major cancers. This article reviews a new laboratory discovery that ultrasound can be used as an antidote for the peripheral cytotoxicity of taxane drugs and discusses the potential development and application of low intensity ultrasound to prevent side effects in chemotherapeutic treatment of cancer patients.

## 1. Cancer chemotherapy: taxanes/paclitaxel

Paclitaxel (brand name Taxol) is a key drug in the current treatment of several major solid tumors, including ovarian cancer 1,2. A paclitaxel dose-dense protocol is also effective to treat recurrent ovarian cancer 3-5. Paclitaxel targets tubulin, and alters the dynamics and stabilizes microtubule filaments 6-8, leading to cell death 9-11. The major side effects (neutropenia, peripheral neuropathy, and alopecia) are thought to be caused by the impact of paclitaxel on the dynamics of the microtubules and killing of mitotic cells 12, including rapidly dividing matrix keratinocytes in the hair follicles 13,14 and the replenishment of neutrophils 15. The interference of paclitaxel on neuronal microtubule dynamic remodeling leads to peripheral neuropathy.

Although generally paclitaxel is highly effective with tolerable side effects, several key side effects include peripheral neuropathy, neutropenia, and alopecia 15. Neutropenia and alopecia are putatively caused by the high proliferative activity of the hematopoietic stem cells and matrix cells of the hair follicles, respectively. The presentation of peripheral neuropathy is assumed because of the critical roles of microtubules in the function and maintenance of neuronal axons.

## 2. Taxane/paclitaxel mechanism of action

Paclitaxel, the first Taxane class of compounds, is a surprisingly successful anti-cancer drug 2,5,16. Paclitaxel was first identified to have cytotoxicity to cancer cells in a search for anti-cancer activity from compounds derived from plants, in paclitaxel's case the Pacific yew tree (*Taxus brevifolia*) 10,12,17. The discovery of its activity to bind and stabilize microtubules 6-8 and consequently inhibit mitosis 9-11 prompted its clinical development.

The traditional view, largely based on studies of paclitaxel on cancer cells in culture, is that paclitaxel binds and stabilizes microtubules, and the key consequences relevant to its efficacy is mitotic inhibition and ultimately apoptosis 8,9,18. Additional studies indicate that slippage in mitotic inhibition and the aberrant mitosis and mitotic catastrophe that result are an important mechanism in the efficacy of paclitaxel treatment 10,19.

However, alternative opinions of a minority contend that paclitaxel also kills cancer cells by a non-mitotic mechanism 20-24, which may be even more important than targeting the proliferative characteristic of cancer cells. Remarkably, paclitaxel killing is p53-independent as high grade ovarian cancer generally has inactivated p53 16, and the cancer cells are commonly insensitive to apoptotic stimulation 19. Several studies also suggest that paclitaxel appears not to directly stimulate the activation of caspase-3, and its efficacy is independent of caspase-3 activation or through a classical apoptosis pathway 19.

Nevertheless, the observations are consistent that microtubules are the specific and relevant drug targets 16. Another observation is that paclitaxel causes the formation of multiple micronuclei without chromatin condensation in cancer cells, a phenomenon coined as “micronucleation”, to distinguish from “nuclear fragmentation” that describes apoptosis and involves chromatin condensation 19. This formation of multiple micronuclei is proposed to be important for cancer killing through the activation of the innate immunity and inflammatory pathway 19.

**Figure 1:**
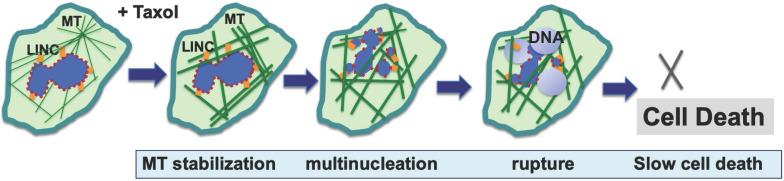
** Proposed mechanisms for paclitaxel-induced breaking of the nuclear envelope and multiple micronucleation in cancer killing**. Based on new studies, a mechanism has been suggested for the anti-cancer activity of paclitaxel: paclitaxel induces disorganized and rigid microtubule (MT) bundles, which apply physical forces to the nuclear envelope through LINC (linker of nuclear and cytoskeleton) bridges, resulting in the breaking of associated malleable nuclei of neoplastic cells and the formation of multiple micronuclei. Paclitaxel induces breaking of nuclear envelope in both mitotic and non-mitotic cancer cells. The micronuclei are defective in membrane structure (illustrated by dotted outline) and have high propensity for rupture and release of chromatin material, resulting in compromised nuclear structure and cell death.

Generally, in the presence of paclitaxel and its interference on microtubule function, the formation of multiple micronuclei likely results from aberrant, multipolar mitosis 10,19. A new study proposes a non-mitotic mechanism for the paclitaxel-stimulated formation of micronuclei, as the rigid microtubule bundles associating with the nuclear envelope physically pull and distort the structure 25. The proposal of a physical force exerted by paclitaxel-induced rigid microtubule filaments in breaking malleable cancer nuclei provides a non-mitotic mechanism to generate multiple micronuclei 24,25 (**Fig. 1**). This proposed mechanism offers a possible alternative explanation for the well-established dogma that paclitaxel targets mitosis in cancer therapy; rather, paclitaxel likely aims at the weakened nuclear envelope of malignant cells. The study provides a new realization that paclitaxel can induce the generation of micronuclei in cells at S-phase by a non-mitotic mechanism 25. In addition, for paclitaxel to target proliferative, mitotic cells, the nuclear envelope malleability appears to be another characteristic that favors cancer versus benign cells. The loss or reduction of nuclear lamina proteins, especially Lamin A/C, in cancer cells has been previously noted 26-29. Thus, malleability of cancer nuclear envelope provides another specificity for paclitaxel, in addition to cell proliferation 24.

Another observation is that a brief treatment of ovarian cancer cells with paclitaxel induced the formation of rare cells with enlarged and deformed nuclei, likely a result of suppression of mitosis but continuous genomic replication 30. These cells, termed “polyploid giant cancer cells” (PGCCs) have been found also to be present in cancer tissues and to harbor characteristics of stem cells found in early embryos 31. An emerging concept, established by a series of publications in the last decade 30-33, proposes that the PGCCs are ovarian cancer stem cells, and they may account for the ability of cancer to gain resistance to chemotherapy 32,33. In a previous study giving similar idea, the paclitaxel-induced multinucleated cells were suggested to associate with paclitaxel resistance 34. Thus, paclitaxel-induced nuclear structural changes have interesting implications in ovarian cancer progenitor cells, mechanisms in paclitaxel cell killing, and drug resistance.

## 3. Retention of paclitaxel within affected cells

The pharmacokinetic properties of paclitaxel in human patients have been documented 35. Paclitaxel is commonly administrated by infusion over several hours. Although a high plasma concentration is reached, the level in blood declines rapidly following infusion, with a half time of just hours 35. Very little paclitaxel or its metabolites are secreted through urine, and sequestration of paclitaxel in tissues/cells is likely a key mechanism in the clearance of the drug 36.

Tumor cells and normal tissues take up and sequester abundant drugs into cells during the infusion, at several hundreds of times the concentration found in the extracellular space 8,18. Paclitaxel binds with high affinity to alpha-tubulin within microtubules at nearly a 1:1 stoichiometry 37, and the concentration of tubulin in cells is calculated to be in the range of 10-20 µM 8,18,38,39. The ability of cells to uptake and concentrate paclitaxel results in part from paclitaxel sequestration by binding to abundant microtubules and tubulins 8,18,38,39. Intracellular paclitaxel is not washed out, but rather retained over several days after exposure, during which time the rigid microtubules persist 8,18,40,41.

**Figure 2:**
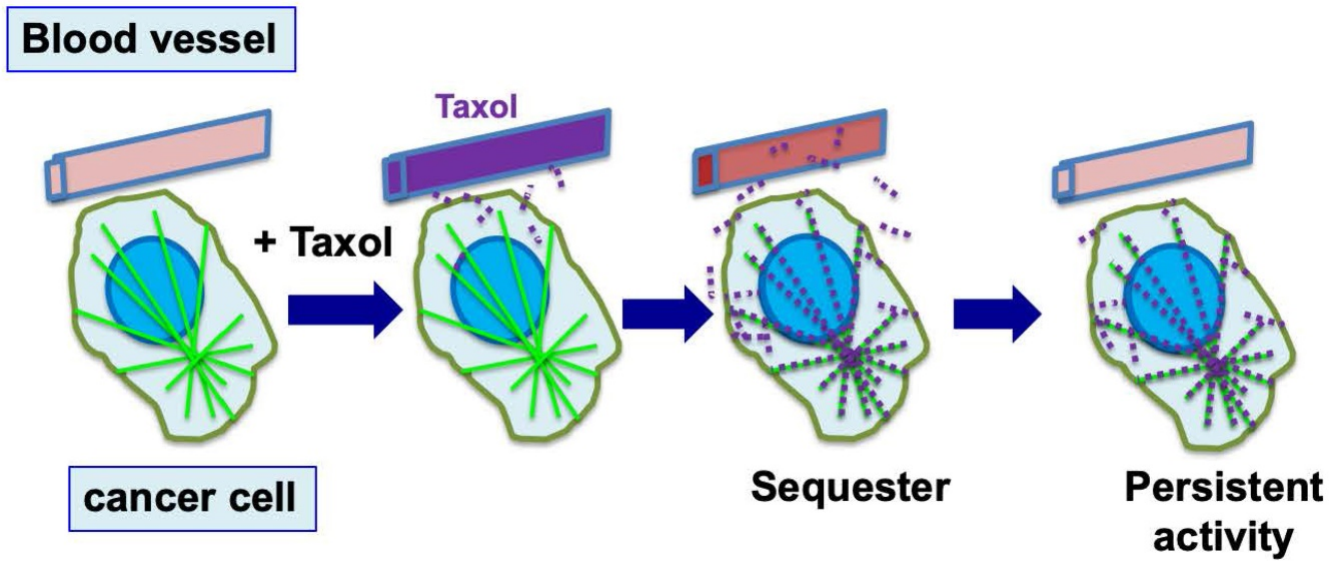
** Cellular sequestration and retention of paclitaxel enables efficient killing of tumor cells.** During chemotherapy, paclitaxel (Taxol) is administrated to patients over 3-6 hours, and reaches a peak concentration in plasma by the end of drug infusion. Over the next 6 to 10 hours, paclitaxel level declines rapidly, and the drug is concentrated in cells (largely by binding to microtubules) several hundred times over the blood level (illustrated by red dots). By binding to microtubules, paclitaxel persists at high levels inside cells for the next 3 to 7 days, where the drug triggers nuclear envelope breakage and the death of cancer cells.

In laboratory studies, paclitaxel was found to be concentrated several hundred-fold into cultured cells 8,37. In animal and patient studies, paclitaxel was found present from several days to a week within cells and tumor tissues, though the drug level had been well cleared in plasma 41,42. Thus, following drug administration, paclitaxel is sequestered and retained within cells by binding to microtubules for a prolonged period of time. Paclitaxel activity persists, resulting in the death of the cancer cells over a few days. However, the persisting activity also causes undesirable side effects (**Fig. 2**). Although it seems to have not been emphasized, this unique property of intracellular paclitaxel retention is likely an important factor for the success of paclitaxel as an anti-cancer drug.

Microtubules are polymers of alpha- and beta-tubulin heterodimers, and play multiple roles in cellular functions 43,44. Microtubules are dynamic: the filaments are constantly extending and shortening, with a balance between the cellular pool of alpha- and beta-tubulin dimers and microtubule polymers, which are about half and half under normal conditions 39,45. Paclitaxel promotes 90-100% of tubulins into polymerized forms 39,45,46.

Tubulins are relatively stable, and the tubulin protein is removed by proteasome- (but not lysosome-) mediated degradation 47 and via degradation by cathepsin D 48. Because of the importance of microtubules in cellular function, the homeostasis of tubulins is tightly regulated 49,50. Tubulins control their synthesis by autoregulation at the mRNA stability 51. Thus, addition of paclitaxel to eliminate alpha- and beta-tubulin dimers (into polymers) stimulates production of new tubulins. Newly synthesized tubulins will further sequester paclitaxel until all available drugs are depleted.

## 4. Taxane side effects: pathology and mechanisms

**Figure 3:**
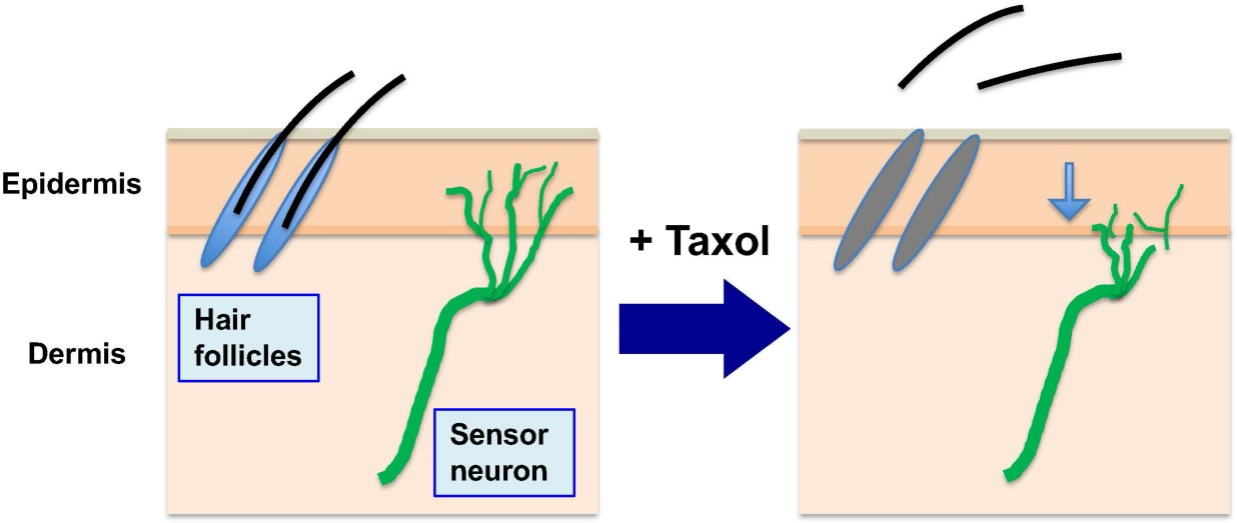
** Alopecia and peripheral neuropathy (in addition to neutropenia) are the main side effects of paclitaxel in chemotherapy.** During chemotherapy to kill cancer cells, paclitaxel (Taxol) is administrated to patients over several hours (3 to 6 hours), and the drug is sequestered and concentrated in both tumor and normal cells (by binding to microtubules). While paclitaxel presented in high levels inside cells triggers death of cancer cells over the next 2-3 days, it also causes death of the proliferative hair matrix keratinocytes, leading to shedding of the hair shaft and alopecia. The retained paclitaxel also damages and causes retraction of sensory neuron terminals, causing peripheral neuropathy. (Noted that neutropenia is another major side effect, not illustrated here).

The common side effects of taxane chemotherapy are neutropenia, peripheral neuropathy, and alopecia 15 (**Fig. 3**). Both neutropenia and peripheral neuropathy are often the dose limiting factors in cancer treatment using taxanes 15, and effective interventional procedures would be highly valuable, but are not available currently. Alopecia, though it may not limit treatment protocol, is nevertheless a distressful quality-of-life issue for many cancer patients and is the top concern voiced by patients during consultations of chemotherapy treatment 52. These side effects are explained by the targeting of microtubules in the host cells, either with roles in mitosis for hematopoietic stem cells or for matrix cells in hair follicles, resulting in neutropenia or alopecia, respectively, or a role in axonal maintenance and function resulting in peripheral neuropathy.

Neutropenia is the principal dose-limiting toxicity of paclitaxel with an early onset (around day 8), when neutrophil counts drop by day 8 to day 11, followed by rapid recovery on days 15 to 21 15. Neutropenia is not cumulative, suggesting that the drug does not permanently damage the hematopoietic stem cells, which seem to be able to recover fully. Obviously, for hematopoietic stem cells, which are one of the most proliferative cell types, inhibition of mitosis by paclitaxel impedes renewal of the immune cells 15. However, the suppression of white blood cells often is transient, and the neutrophil level often can recover in a few days 15. Additionally, the issue when presented is routinely managed by giving granulocyte colony stimulating factor (G-CSF) to enhance the expansion of hematopoietic stem cells 15.

Another highly proliferative cell type is the matrix cells of the hair follicles. Hair follicles undergo cycles of growth (anagen), regression (catagen), and relative quiescence (telogen) throughout life 53. In humans, normally 85~90% of scalp hair follicles are in anagen at any given time, when hair follicle matrix cells undergo rapid proliferation, making them extremely susceptible to mitotic inhibitors such as paclitaxel. Paclitaxel induces massive mitotic defects and apoptosis in transit amplifying hair matrix keratinocytes and also severely damages epithelial stem/progenitor cells in the bulge and outer root sheath of human scalp hair follicles 14. This results in hair shedding that begins as early as 1-3 weeks after initiation of chemotherapy 13,54. More than 80% of patients receiving paclitaxel develop alopecia, and beard, eyebrows, and eyelashes can also be affected, in addition to scalp hair. Hair regrowth usually takes 3~6 months after cessation of chemotherapy, but in a substantial number of cases, hair loss is irreversible 52,55. While killing of mitotic hair matrix keratinocytes accounts for the drastic hair loss, direct epithelial stem cell damage likely causes irreversible hair loss 14.

Sensory neuropathy, presenting as numbness and pain of feet and hands, is often the dose-limiting toxicity of the taxane agents 12,15,56,57. In this case, mitosis is not the target in this terminally differentiated cell type. However, microtubules play critical functions in neuronal axons, and paclitaxel targeting would cause a pathological situation. This major side effect (peripheral neuropathy) is thought to be caused by the impact of paclitaxel on the dynamics of the axonal microtubule structure, presumably altering the distribution and structure of the peripheral neuron network and the transmission of nerve stimuli 12,58. Clinical features of peripheral neuropathy often present with a reduced density of peripheral neuron fibers, visualized using diagnostic ultrasound 56,59,60(**Fig. 3**). Extensive laboratory research using cell systems, nonmammalian animal models, and rodent models has been attempted and is ongoing to understand the complex mechanisms of paclitaxel in causing peripheral neuronal damage 61-63. Paclitaxel causes neurite retraction in these cultured neuronal cells, and it is considered that the phenomenon models the neuronal damage in paclitaxel-induced peripheral neuropathy 12. Although the binding and stabilization of neuronal microtubule bundles by paclitaxel is thought to be the initial cause, the subsequent pathways and mechanisms leading to neuronal damage are complex and remain unresolved.

## 5. Research efforts to counter paclitaxel/taxane side effects

Oncologists have invested substantial research effort to find strategies to counter the side effects and fully realize the power of taxanes in the treatment of a wide range of cancer types 15. Developing a practical strategy to prevent the side effects of paclitaxel is indeed a difficult problem to solve, and various models have been used to investigate 63. Potential drugs to block paclitaxel cytotoxicity will also inhibit the cancer killing activity, making the potential drugs unusable. Many studies tested agents with alternative mechanisms, though so far none have been found to reduce the symptoms 63,64.

The underlying pathobiology of paclitaxel chemotherapy-induced alopecia remains poorly understood. Recently it was shown that paclitaxel induces massive mitotic defects and apoptosis in transit amplifying hair matrix keratinocytes and also severely damages epithelial stem/progenitor cells in the bulge and outer root sheath of human scalp hair follicles 14. This newly identified damage directly to stem/progenitor cells likely explains the severity and permanence of paclitaxel-induced alopecia. Since paclitaxel, as a mitotic inhibitor, targets cells at mitosis, or the M phase of the cell cycle 10,11,65, blocking of hair matrix cells from entering into mitosis is a potential strategy to prevent hair follicle damage and possibly alopecia. Indeed, using the CDK4/6 inhibitor palbociclib to keep cells of human hair follicles in organoid culture in G1, paclitaxel-induced apoptosis (shown by caspase-3 activation) is prevented 14. This concept has been applied to test CDK4/6 inhibition for the protection against chemotherapy-induced acute kidney injury 66,67 and chemotherapy-induced hematopoietic stem cell exhaustion 68. However, systematic blocking of mitosis likely will also reduce efficacy of paclitaxel activity in chemotherapy. Additionally, the CDK4/6 inhibitor itself already presents alopecia as a side effect 69-71. Thus, the possibility of using the CDK4/6 inhibitor to prevent paclitaxel-induced alopecia may require fine calibration of drug dosage and drug administration sequence and schedule.

In addition to neutropenia, which is often transient and readily recoverable, severe peripheral neuropathy is accumulative and is often the dosage limiting factor in treatment using taxane drugs 15,36. In patient biopsies and animal models, paclitaxel treatment was observed to induce reduction of sensory neuron ends 63. *In vitro* studies indicate that paclitaxel causes neurite retraction and neuron cell degeneration, leading to the idea that paclitaxel targeting of axon microtubules is the cause of neuronal retraction and peripheral neuropathy 58,72. However, some studies indicate peripheral neuropathy may precede axon retraction 73,74. Although the initial step appears to be the paclitaxel binding to axonal microtubules and their stabilization, downstream pathways leading to the pathology are more complex 63.

Many studies and various proposed mechanisms have been reported 63. As an example, studies propose that the damage to epidermal mitochondria and generation of H_2_O_2_, and subsequent activation of MMP-13, are involved in neurite degeneration 62,75. Upregulation of MMP-13 by paclitaxel has also been confirmed in mammals to be a potential mechanism for paclitaxel-induced peripheral neuropathy 62,75. MMP-13 is a member of the matrix-metalloproteinase family of matrix-degrading enzymes, and its suppression likely will not affect the activity of paclitaxel in killing cancer cells. Thus, pharmacologic inhibition of MMP-13 may be a potential strategy to prevent paclitaxel-induced peripheral neuropathy 62,75.

Despite their recognized importance and the extensive efforts devoted to the issues related to paclitaxel side effects, to date, few approaches are practical and available to counter taxane side effects in chemotherapy 63. The mechanisms of paclitaxel induced neuronal damage and development of peripheral neuropathy have been extensively explored, and many drugs and agents have been suggested to have neuroprotective effects in basic laboratory studies. Some of these drugs have been tested in clinical studies for their protective effects. In the pre-clinical research, these neuropathy inhibitors are proposed to have mechanisms targeting oxidative stress, inflammatory response, ion channels, transient receptor potential channels, cannabinoid receptors, and the monoamine nervous system. However, very few drugs have demonstrated any efficacy in protecting paclitaxel-induced peripheral neuropathy in clinical trials 64, and none has reached clinical utility.

## 6. Prevention of taxane chemotherapy side effects using cryo-treatment

Currently, no satisfactory methods are available to reverse the side effects of paclitaxel, though cooling of hands and feet to limit drug exposure has been studied as a possible strategy to limit peripheral neuropathy of hands and feet 76,77. For protection from hair loss, only scalp cooling has been established as a method to limit drug exposure to the scalp and to prevent or reduce alopecia 78, but its success is limited and unpredictable 79,80, and scalp cooling caps cannot protect eyebrows, eyelashes and facial hair. Therefore, novel approaches to prevent paclitaxel-induced alopecia are urgently needed to improve the quality of life of cancer patients.

The cooling needs to be maintained during the entire paclitaxel infusion period, generally 3 to 6 hours 35. Cooling hands and feet with cooling gloves and socks, or the scalp with a cold hat/cap, works to limit the blood flow and thus the exposure to drugs. However, possible mechanisms such as the impact of temperature on microtubules polymerization, and slowing the rate and number of mitotic hair follicle matrix keratinocytes that enter mitosis, may also contribute to the outcome.

## 7. Discovery that low intensity ultrasound to be an antidote for paclitaxel cytotoxicity

The new discovery that low intensity ultrasound is an antidote of paclitaxel cytotoxicity 81 may offer a unique and effective strategy to eliminate the side effects of paclitaxel in cancer chemotherapy.

Ultrasound technologies have extensive applications in medicine, either for diagnosis (sonogram) or therapy 82-84. Typically, ultrasound with extremely low intensity (1-50 mW/cm^2^) and high frequency (such as 50 MHz) is used for diagnostic (imaging) purposes. High intensity (> 8 W/cm^2^, 20-60 kHz) ultrasound that can deliver strong energy is used for surgery and disruption through heating and acoustic cavitation. The medical application of ultrasound with an intensity that is low yet sufficiently high to produce biological activity is known as ultrasound physiotherapy 83,84, which uses sufficiently strong but non-disruptive ultrasound shock waves (0.5-3.0 W/cm^2^). The most commonly used devices produce ultrasound waves with frequencies either around 1-3 MHz or 20-150 kHz (known as long wavelength ultrasound). Similar effects by either 1-3 MHz or 45 kHz ultrasound waves on cells and tissues were reported in several studies 85,86. The majority of ultrasound for physiotherapy uses frequencies in the range of 1-3 MHz, which traditionally is thought to produce less cavitation and thus less tissue damage. However, more recent laboratory findings indicate that the low frequency (20 to 100 KHz) ultrasound seems to produce a stronger biological impact 82,83,87,88, and at the same time seems to produce no cell or tissue damage 87,89,90. With the availability of efficient low frequency ultrasound devices 91, low frequency ultrasound has found suitable application in several medical procedures 82-84.

**Figure 4:**
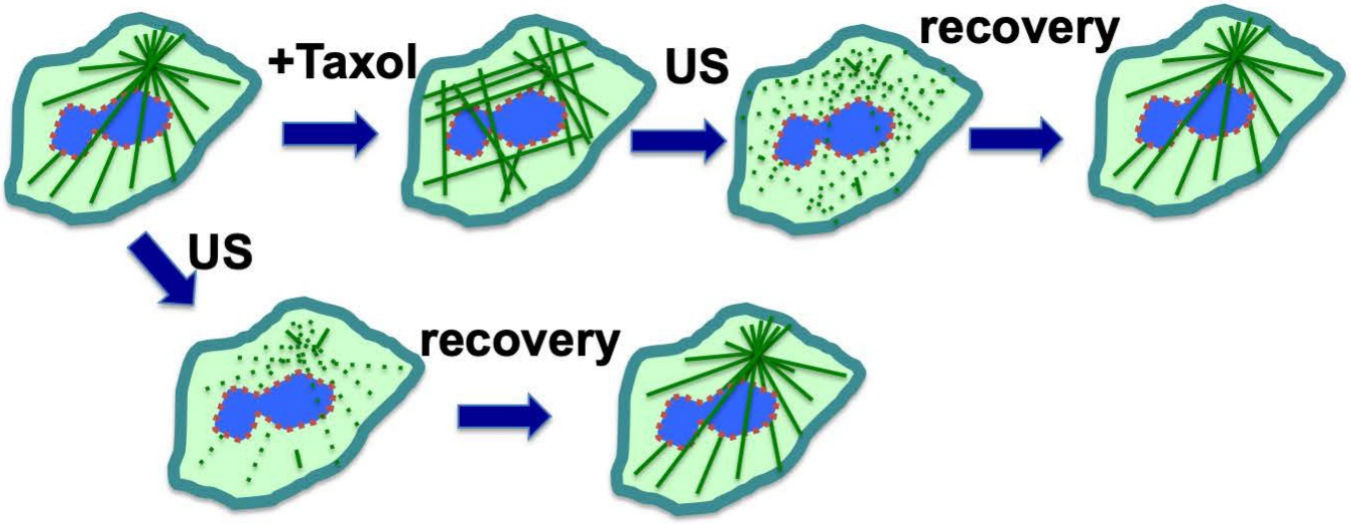
** Ultrasound exposure reverses cytotoxicity by disrupting rigid microtubule filaments induced by paclitaxel (Taxol) treatment of cells.** Microtubule bundles radiate out from the microtubule organizing center. Ultrasound (US) is known to transiently disrupt microtubule networks, which reform within 1-2 hours. Paclitaxel (Taxol) induces rigid microtubule filaments that lead to growth arrest and subsequent cell death in proliferative cells such as cancer cells or matrix keratinocytes of the hair follicles. We suggest a mechanism through which ultrasound reverses cytotoxicity by disrupting rigid microtubule filaments induced by paclitaxel. The paclitaxel-bound microtubule fragments and tubulins are relocated to lysosomes for degradation, and newly synthesized tubulins form a new network of microtubule cytoskeleton without bound paclitaxel. Thus, a brief exposure to low intensity ultrasound removes cellular paclitaxel activity/cytotoxicity.

A serendipitous discovery is that ultrasound can eliminate paclitaxel cytotoxicity in cells. Although exposure to low intensity ultrasound (1 W/cm^2^) alone, for up to 10 minutes, had no noticeable impact on cultured cells, it actually reversed the cytotoxicity of paclitaxel in cancer cells 81. Ultrasound at low intensity can disrupt microtubule cytoskeleton transiently without significant impact on cell survival 92,93. For cells in culture, paclitaxel/Taxol treatment resulted in the appearance of strong staining of microtubule filaments, which was abolished by low intensity ultrasound (**Fig. 4**) 81. After treatment with ultrasound and recovery, the microtubule cytoskeleton appeared to have the same morphology in paclitaxel-treated cells as those without paclitaxel treatment (**Fig. 4A**), but the ultrasound exposure completely eliminated paclitaxel cytotoxicity (**Fig. 4**) 81. The finding was repeated and confirmed in various cell types, and it was concluded that low intensity ultrasound is capable of eliminating paclitaxel induced cytotoxicity in all cell types tested, by transiently breaking the rigid microtubule filaments 81 (**Fig. 4**).

Based on these results, a concept was developed that ultrasound reverses cytotoxicity by disrupting rigid microtubule filaments induced by paclitaxel treatment of proliferative cells (**Fig 4**) 81. The physical breakage of paclitaxel-bound microtubules by ultrasound shock waves will result in the relocation of paclitaxel-bound microtubule fragments or tubulin heterodimers to lysosomes for degradation, and new microtubule networks will form rapidly from tubulins not bound to paclitaxel 81. Tubulin levels in cells are auto-regulated, and newly synthesized tubulins quickly replace degraded paclitaxel-bound tubulins to form the microtubule cytoskeleton 49,51,94. Thus, a brief pulse exposure to ultrasound efficiently removes the negative impact of paclitaxel on microtubule dynamics and cell cytotoxicity (**Fig. 4**).

A potential strategy is to reverse the early step of paclitaxel cytotoxicity by disrupting the rigid microtubule filaments induced by paclitaxel with low intensity ultrasound. Thus, the strategy may be successful to counter paclitaxel-induced peripheral neuropathy at an early step, regardless of the complex down-stream mechanisms by which paclitaxel induces peripheral neuropathy.

**Figure 5:**
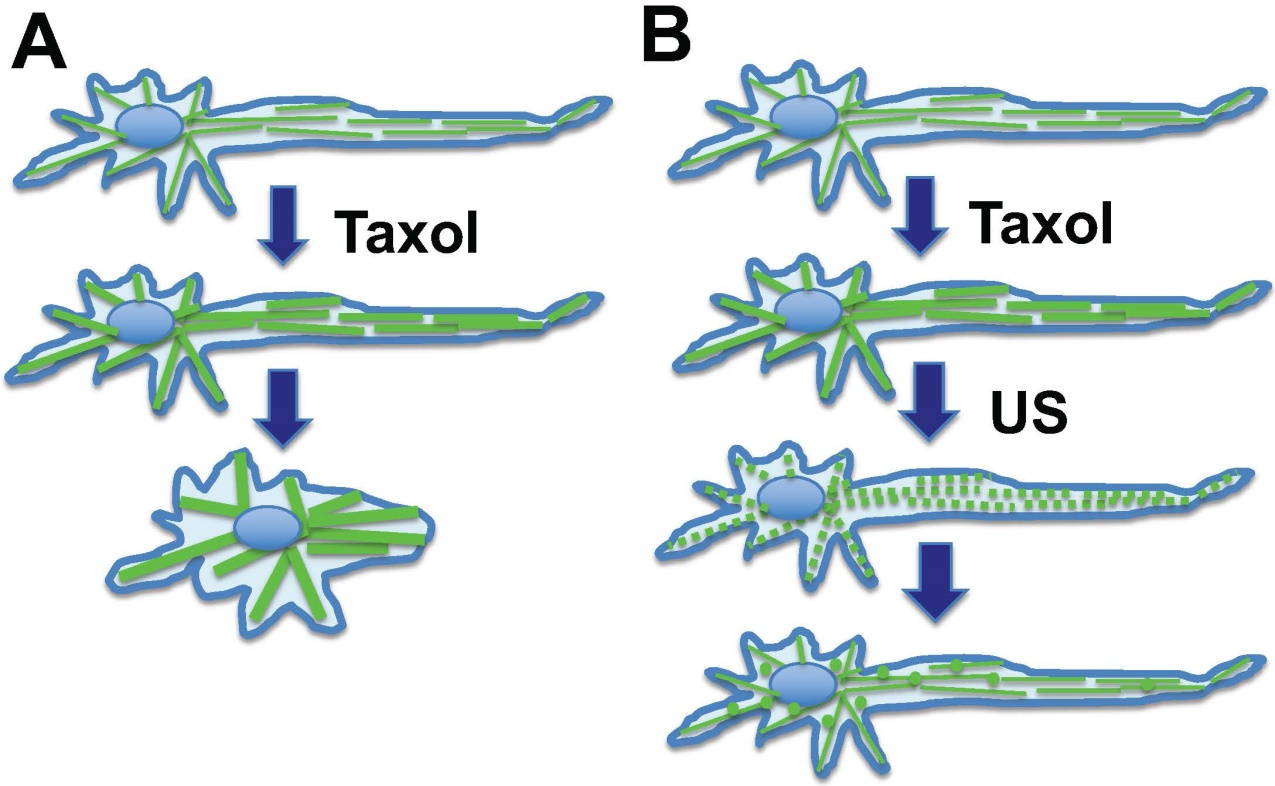
** Hypothesis: Ultrasound reverses neurite retraction by disrupting rigid microtubule filaments induced by paclitaxel.** Axon maintenance in peripheral neuronal cells depends on microtubule dynamics. (**A**) Taxol/paclitaxel stabilizes microtubules and leads to neurite retraction. This may model the underlying issue of paclitaxel-induced peripheral neuropathy. (**B**) Ultrasound (US) is known to disrupt the microtubule network, which in turn reforms rapidly. A mechanism is suggested that ultrasound disrupts paclitaxel-induced rigid microtubule bundles, leading to prevention of neurite retraction, and this concept may be explored to prevent paclitaxel-induced peripheral neuropathy.

In chemotherapy, paclitaxel also causes stabilization and bundling of microtubules in peripheral neuronal cells, and consequently leads to retraction (**Fig. 5A**). This may reflect the underlying issue in peripheral neuropathy, which is thought to be caused by the impact of paclitaxel on the dynamics of the axonal long microtubule structure, presumably altering the distribution and structure of the peripheral neuron network and the transmission of nerve stimuli 63,95. Low intensity ultrasound transiently disrupts the paclitaxel-induced rigid microtubule bundles (**Fig. 5B**). The paclitaxel bound microtubule fragments undergo lysosomal degradation. Additionally, paclitaxel is rapidly (8-24 hours) eliminated in the environment by binding to and being sequestered by cellular microtubules (about 10-20 µM in cells) 38,39,42, or bound by other cellular components. Increased synthesis of tubulins likely also contributes to paclitaxel elimination.

## 8. Potential prevention of taxane chemotherapy side effects using low intensity ultrasound

Low density ultrasound has been explored to influence peripheral neurons 96 or treat pain and neuropathy in clinical trials 97; however, the current concept is based on a new discovery that ultrasound can eliminate acute paclitaxel cytotoxicity by breaking the rigid microtubules. While low intensity ultrasound has been extensively used for various medical applications 83,84, and laboratory studies show that ultrasound waves impact cells and may have biological activity even on hair follicles 98, the evidence to support medical applications of ultrasound is largely anecdotal. Most medical applications of ultrasound physiotherapies were determined to lack true merit in large and rigorous clinical studies 83,84. Instead, the surprising findings 81 and unique hypothesis discussed here may be possible to introduce a rational, evidence-based use of ultrasound therapy for overcoming paclitaxel cytotoxicity into medical practice.

**Figure 6:**
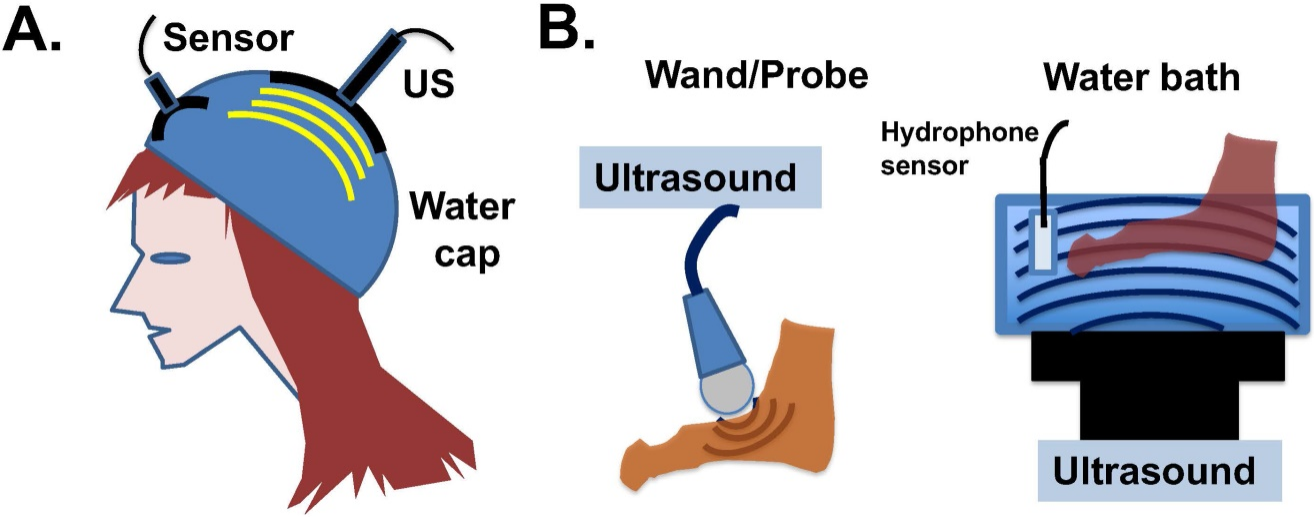
** Potential application of ultrasound devices for physiotherapy to prevent paclitaxel side effects in chemotherapy.** (**A**) Illustration of ultrasound design to counter cytotoxicity in hair follicles of cancer patients undergoing paclitaxel (Taxol) treatment. A water-filled bag/cap is used to couple the transfer of ultrasound energy to epidermal and hair follicles under the wetted hair. Ultrasound intensity will be monitored (and potentially feedback regulated) by a built-in hydrophone sensor. (**B**) Illustration of two available devises for the transmission of ultrasound waves through water to peripheral tissues. The water bath ultrasound device gives out 45 kHz ultrasound shock wave with 1 to 3 W/cm^2^ energy, and the probe ultrasound device produces 1 to 3 MHz ultrasound in 1 to 3 W/cm^2^ energy waves.

Since ultrasound treatment is considered a safe procedure, potential clinical development is eminently feasible. Ultrasound devices suitable to apply low intensity shock waves to the skin surface and hair follicles may be designed, and water may be suitable to be used as an ultrasound-transmitting medium (**Fig. 6A**). The human scalp is a strong barrier for the penetration of low frequency ultrasound energy 84, which actually eases the potential concern that the ultrasound waves may affect human brain. The application of ultrasound to hands, feet, and scalp a few hours after drug administration may be able to eliminate the persistent activity of paclitaxel already bound to the microtubules of the cells. Both the water bath and probe types of ultrasound devices can be considered for clinical application (**Fig. 6B**). The bath device produces 45-150 kHz, 1-3 W/cm^2^ ultrasound waves with adjustable frequency and energy levels. The probe device produces adjustable 1-3 W/cm^2^ and 1-3 MHz ultrasound waves.

**Figure 7:**
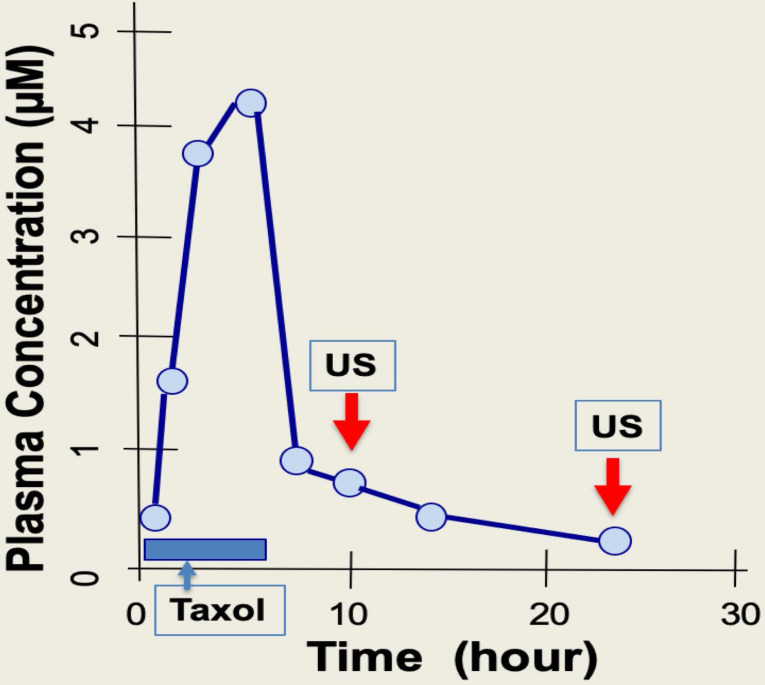
** Timing of ultrasound application for physiotherapy to prevent paclitaxel side effects in chemotherapy.** During chemotherapy, paclitaxel (Taxol) is administrated to patients over 3-6 hours, and taxane concentration reaches a peak level in plasma by the end of drug infusion. Paclitaxel plasma levels fall rapidly following infusion over next 6 to 10 hours. It is reasoned that intermittent ultrasound (US) pulse treatment (5 min exposure) over a period of 4-10 hours may be suitable, when plasma drug levels are much lower. A second ultrasound treatment at around 24 hours may also helpful to further eliminate paclitaxel cytotoxicity locally at hands, feet, and scalp.

Paclitaxel is administrated through IV over a 4- to 6-hour time course. Upon completion of drug infusion, blood paclitaxel rapidly declines, and the drug enters and accumulates within cells 8,35,42. The cellular level of paclitaxel is concentrated several hundred-fold higher than in blood, partly due to binding to cellular microtubules 8,35,37. A short exposure of cells to paclitaxel leads to persistent cytotoxicity over several days even when extracellular sources of paclitaxel is absent, as the paclitaxel bound rigid microtubule bundles are present several days after 41. During the 2-7 days after paclitaxel administration, the drug triggers death of cancer cells, but also causes damage to peripheral neurons that presents as peripheral neuropathy, and also to hair matrix keratinocytes that results in alopecia 14. Thus, for a 3- to 6-hour infusion of paclitaxel, it may be suitable to treat patients with intermittent, low intensity ultrasound pulse therapy (5 min, about 1 W/cm^2^) over just a few hours (e.g. 4 to 10 hours) following chemotherapy (**Fig. 7**). Possibly, a second ultrasound treatment may be applied 24 hours after chemotherapy to ensure complete localized elimination of paclitaxel cytotoxicity (e.g., in scalp skin but not in cancer cells) (**Fig. 7**). The optimal timing of ultrasound exposure to reverse paclitaxel activity may be the window between the time when free paclitaxel is depleted, and the time required for causing damage and harm by the paclitaxel-bound rigid microtubules (**Fig. 7**). Thus, ultrasound treatment appears to disrupt the persistent paclitaxel-bound rigid microtubules and thereby eliminate the prolonged cytotoxicity of paclitaxel.

Newer microtubule stabilizing drugs and delivering platforms, such as Abraxanes, cabazitaxel, epothilones (ixabepilone), laulimalide, and discodermolide, etc., are under development and coming into clinical application 99-104. These new agents act with a mechanism same as paclitaxel, the first generation of microtubule stabilizing drugs, with similar side effects. Similarly, low intensity ultrasound likely will disrupt microtubules affected by these new drugs in the same way the ultrasound acts on paclitaxel-induced rigid microtubule bundles. Thus, ultrasound treatment may also relieve side effects from the new microtubule stabilizing drugs.

The application of ultrasound to counter paclitaxel-induced peripheral neuropathy seems to be very feasible and practical. In fact, probe ultrasound has been used in clinical trials to treat paclitaxel-induced peripheral neuropathy 97. However, the current research findings suggest that ultrasound may prevent, but not reverse paclitaxel-induced peripheral neuropathy and alopecia, and this will be a new rationale based on a solid scientific basis.

## 9. Summary

Paclitaxel (and other taxane drugs) binds and stabilizes microtubules, while ultrasound breaks microtubules, thus acting as an antidote. The mechanism may be quite self-explanatory. Based on our recent discovery that low intensity ultrasound treatment can effectively and almost completely neutralize the cytotoxic effects of paclitaxel in various cell types, a strategy may be developed to counter the side effects of paclitaxel-based chemotherapy for cancer patients. The concept that a brief exposure of paclitaxel-treated cells to ultrasound is sufficient to break paclitaxel-induced rigid microtubules opens up the possibility to use ultrasound locally to eliminate cytotoxicity at only the desirable anatomic sites, without affecting paclitaxel activity towards neoplastic cells. In essence, ultrasound is able to remove the persistent activity of paclitaxel at intended areas after systematic drug infusion. The possibilities to use this paclitaxel antidote can be contemplated to prevent chemotherapy-induced alopecia, and peripheral neuropathy, which will ensure the full use of paclitaxel dosage in treatment and improve the quality of life for cancer patients.
